# 87. Machine Learning-Guided Generation of a Potent Combination of Broadly Neutralizing Sarbecovirus Antibodies with a High Barrier to Resistance

**DOI:** 10.1093/ofid/ofae631.024

**Published:** 2025-01-29

**Authors:** Kimberly Schmitt, Grace Marden, Eric Carlin, Alex Ramos, Hongru Li, Nadine Shaban, Monica Menzenski, Geetika Sharma, Ross Federman, Alan Leung, Nicole Leon Vargas, Brett T Hannigan, Pamela Russell, Adam Root, Heather A Van Epps, Kristen Hopson, Amarendra Pegu, Gevorg Grigoryan, Gavin CKW Koh, Daria Hazuda, Francesco Borriello

**Affiliations:** Generate:Biomedicines, Somerville, MA; Generate Biomedicines, Quincy, Massachusetts; Generate Biomedicines, Quincy, Massachusetts; Generate Biomedicines, Quincy, Massachusetts; Generate Biomedicines, Quincy, Massachusetts; Generate Biomedicines, Quincy, Massachusetts; Generate Biomedicines, Quincy, Massachusetts; Generate Biomedicines, Quincy, Massachusetts; Generate Biomedicines, Quincy, Massachusetts; Generate Biomedicines, Quincy, Massachusetts; Generate Biomedicines, Quincy, Massachusetts; Generate Biomedicines, Quincy, Massachusetts; Generate Biomedicines, Quincy, Massachusetts; Generate Biomedicines, Quincy, Massachusetts; Generate Biomedicines, Quincy, Massachusetts; Generate Biomedicines, Quincy, Massachusetts; Generate Biomedicines, Quincy, Massachusetts; Generate Biomedicines, Quincy, Massachusetts; Generate:Biomedicines, Somerville, MA; Generate Biomedicines, Quincy, Massachusetts; Generate Biomedicines, Quincy, Massachusetts

## Abstract

**Background:**

The elderly and immunocompromised remain at high risk of severe COVID-19 due to suboptimal immune responses. Monoclonal antibodies to SARS-CoV-2 spike were highly effective for both prophylaxis and treatment, but quickly became ineffective due to viral escape. Using a machine learning-guided optimization approach, we identified two antibodies against highly conserved epitopes, one targeting the spike S2 stem helix (GB-0669) and the other against the class 4 RBD site on S1 (PRO-37587) with the goal of developing a combination for prophylaxis that would be less susceptible to viral escape.

**Methods:**

GB-0669 and PRO-37587 were characterized for breadth through binding and neutralization assays against a panel of SARS-CoV-2 variants and related sarbecoviruses. We also assessed the barrier to resistance, both individually and in combination using SARS-CoV-2 Omicron BA.1 in live virus escape studies in Vero E6 cells. Passaged viruses were sequenced and assessed for viral replication. The prevalence of any observed mutations was analyzed for conservation in the SARS-CoV-2 lineages and across the sarbecovirus family.

**Results:**

Both antibodies exhibited potent neutralization across SARS-CoV-2 variants including those that had not yet emerged during development of our antibodies, while displaying activity against non-SARS-CoV-2 viruses. During passage with BA.1, escape from GB-0669 was observed in passage 3 (H1156Y), while escape from PRO-37587 emerged in passage 10 through two mutations (P381S, D402G) that impaired viral replication. By the end of passage 10, neither complete nor partial escape were observed for the combination of GB-0669 and PRO-37587. These escape mutations were observed in fewer than 1% of circulating variants from the beginning of the pandemic, probably due to low selective pressure and/or their impact on viral replication. With the exception of P381S, these mutations were not observed across a range of sarbecoviruses.

**Conclusion:**

We demonstrate that the combination of GB-0669 and PRO-37587 has potent and broad neutralization activity. The robust viral escape profile and conservation of these epitopes suggest that this combination is more likely to remain effective in the face of ongoing viral evolution.

**Disclosures:**

**Alex Ramos, PhD**, Generate Biomedicines: Stocks/Bonds (Private Company) **Alan Leung, PhD**, Generate Biomedicines: Stocks/Bonds (Private Company) **Brett T. Hannigan, PhD**, Generate Biomedicines: Employee|Generate Biomedicines: Stocks/Bonds (Private Company) **Adam Root, MS, MBA**, Generate Biomedicines: Grant/Research Support|Generate Biomedicines: Stocks/Bonds (Private Company) **Heather A. Van Epps, PhD**, Generate Biomedicines: Stocks/Bonds (Private Company) **Kristen Hopson, Ph.D.**, Generate Biomedicines: Stocks/Bonds (Private Company) **Gevorg Grigoryan, PhD**, Generate Biomedicines, Inc.: Employee and shareholder|Generate Biomedicines, Inc.: Stocks/Bonds (Private Company) **Gavin CKW Koh, PhD**, AstraZeneca: Stocks/Bonds (Public Company)|Generate:Biomedicines: Stocks/Bonds (Private Company)|GlaxoSmithKline: Stocks/Bonds (Public Company) **Daria Hazuda, PhD**, Generate Biomedicines: Stocks/Bonds (Private Company)

